# CHILDHOOD SES AND AGE-RELATED BRAIN CHANGES RACIALLY-ETHNICALLY DIVERSE OLDER ADULTS

**DOI:** 10.1093/geroni/igz038.1998

**Published:** 2019-11-08

**Authors:** Indira C Turney, Miguel Arce Rentería, Anthony G Chesebro, Juliet M Colon, Nicole Schupf, Richard P Mayeux, Adam M Brickman, Jennifer J Manly

**Affiliations:** Columbia University Medical Center, New York, New York, United States

## Abstract

Socioeconomic disadvantages in childhood has been linked to dementia in late life. However, the underlying pathways through which childhood socioeconomic status (CSES) affects health in old age is unclear. CSES has been linked to age-related differences in regions affected by Alzheimer’s disease (AD; e.g., hippocampus). CSES varies across race/ethnicity; It is critical to examine the relationship between CSES and age-related brain structural changes across diverse aging populations. We used an established proxy for CSES, number of siblings (i.e., sibship size), to examine whether CSES buffered age-related changes in hippocampal volume in a community-based sample of racially/ethnically diverse older adults. Sibship size moderated age-related differences in hippocampal volume in Whites (β=-5.61[-11.09,-0.12]), but not in Blacks and Hispanics. Results indicate that Whites with no sibling (vs. Whites with siblings) show less age-related difference in hippocampal volume. Future analyses will examine other CSES factors (i.e., parental education/occupation) on age-related structural changes across race/ethnicity.

